# Supplementary material for: The impact of international megaproject social responsibility on satisfaction with the environmental compensation mechanism: The role of stakeholder participation
Source: PLoS One. 2025 Oct 9;20(10):e0334291. doi: 10.1371/journal.pone.0334291 (PMC12510660; doi:10.1371/journal.pone.0334291)
Supplement: S2 File — (DOCX) [file pone.0334291.s002.docx]

**泰国背景下中国承包商重大工程项目社会责任**

**Strategic Management Model for Megaproject Social Responsibility in Supply Chain: The Chinese International Contractor Perspective Operating in Thailand.**

******本问卷分为中文和英文两种语言，请根据自己的情况阅读，感谢您的配合。
**** This questionnaire is divided into two languages, Chinese and English, please read according to your own situation. Thanks for your cooperation.**

尊敬的先生/女士：
您好！本人目前在泰国那黎宣大学在读博士，研究的方向为“在泰国背景下中国承包商重大工程社会责任战略模型”，我需要您的帮助来完成关于重大工程社会责任战略模型的研究！
我国是世界上首屈一指的重大工程建设大国，在重大工程关键技术、组织实施、工程战略资源整合等方面取得了重大成果。但是，随着全球性的社会、环境、工程复杂性等问题的出现，人类命运共同体已经成为前所未有的严峻挑战。为了在泰国的环境背景下对这一问题进行研究，我们设计了在这份调查问卷。
本问卷的问题没有对错之分，您在填写的过程中无需署名，我们将秉承科研人员对学术研究的客观、严谨等特质对您的答卷进行保密。您可能需要15分钟左右的时间进行填写，每一项对我们的研究来说都非常的重要，再次深深感谢您的配合与理解！

问卷说明：
社会责任是指企业在创造利润、对股东承担法律责任的同时，还要承担对员工、消费者、社区、环境以及尊重当地文化的责任，强调在生产的过程中对人的价值的重视，强调对环境、消费者、社会的贡献。
本研究从重大工程的角度出发，在泰国的背景下，重在各参与方所超越组织目标而履行的社会任务。针对项目生命周期的不同以及每个生命周期利益相关者的不同，分别从经济、法律、伦理道德、政治等方面考察项目生命周期各参与方对社会的义务。
由于重大工程社会责任的高度复杂性，问卷设计好之后，得到泰国数名专家的建议，进行了简化处理后选取了相对重要的指标。请在填写问卷时，根据个人感知和意识对每个条目进行重要性或认同度判断。

Dear Sir/Madam,
Hello! I am currently a Ph.D. candidate in Naresuan University, Thailand. My research direction is "Megaproject Social Responsibility Strategy Model of Chinese Contractors in the Context of Thailand". I would like to ask for your assistance to complete the research on social responsibility strategy model of megaprojects.
As the world's leading megaproject construction country, China has made great achievements in key technology, organization and implementation of major projects, and integration of project strategic resources. However, with the emergence of global social, environmental and engineering complexity issues, it has become an unprecedented challenge to focus on the community of shared future for mankind. In order to conduct research on this issue in the context of Thailand, we designed this questionnaire.
There are no right or wrong questions in this questionnaire. You do not need to sign your name when you fill in the questionnaire. We will keep your answers confidential in accordance with the objectivity and rigor of scientific research staff. You may need 15-25 minutes to fill in this questionnaire. Each item is very important for our study. Thank you again for your cooperation and understanding!

Questionnaire Description:
Social responsibility means that while creating profits and assuming legal responsibilities to shareholders, an enterprise should also assume responsibilities for employees, consumers, communities, the environment and respect for local culture. It emphasizes the attention to human value in the process of production and the contribution to the environment, consumers and society.
From the perspective of major projects, in the context of Thailand, this study focuses on the social tasks performed by the participants beyond the organizational goals. In view of the difference of project life cycle and the difference of stakeholders in each life cycle, the obligations of each participant in project life cycle to society are investigated from the aspects of economy, law, ethics, politics, etc.
Due to the high complexity of social responsibility of major projects, the questionnaire was designed, and several experts from Thailand advised to simplify the process and select relatively important indicators. When filling in the questionnaire, please judge the importance or identity of each item according to your personal perception and consciousness.

1. 个人信息
请根据实际情况填写您的个人信息。

1. Demographic Information
Please Please fill in your personal information according to the actual situation.

1.1 您的性别?
Your gender is? [单选题] *

| ○男 Male |
| --- |
| ○女 Female |

1.2 您是否有宗教信仰？
What's your religion [单选题] *

| ○佛教 Buddhism |
| --- |
| ○伊斯兰教 Islam |
| ○基督教 Christian |
| ○无宗教信仰 No Religion |
| ○其他 Other |

1.3 您在大型项目行业工作了多少年?
How many years have you worked in the megaproject industry? [单选题] *

| ○1-5 年 years |
| --- |
| ○6-10 年 years |
| ○大于10年 More than 10 years |
| ○我不从事该行业 I'm not in that line of work |

1.4 您的最高学历是?
Please specify your highest education?
[单选题] *

| ○博士 Ph.D |
| --- |
| ○硕士 Master |
| ○学士 Bachelor |
| ○大专 Diploma/College |
| ○高中 High school |
| ○高中以下 Under the high school |

1.5 您对社会责任熟悉吗?
Are you familiar with social responsibility?
[单选题] *

| ○从来没有听说过 Never heard at all |
| --- |
| ○有一点了解 A little understanding |
| ○有清晰了解 Have clear understanding |
| ○知道的较多 Know more about this |
| ○非常熟悉 Very familiar |

1.6 您是通过什么渠道学习到社会责任的?
How did you learn about social responsibility? [多选题] *

| □大众媒体，如报纸和电视 Mass media such as newspapers and television |
| --- |
| □网络 Internet |
| □培训与学习 Training and learning |
| □社交场合 Social occasions |
| □其他 Others |

1.7 您在参与大型工程项目当中属于以下哪个角色？
For megaprojects, which of the following groups do you belong to? [单选题] *

| ○中国承包商/分包商 Chinese contractor or Sub-contractor |
| --- |
| ○中央政府或地方政府 The central government or local government |
| ○设计方 Designer |
| ○分包商 sub-contractor |
| ○项目法人 project legal |
| ○供应商 supplier |
| ○项目监理 project supervision |
| ○非营利性组织 NGO |
| ○媒体 media |
| ○社区群众 community or public |

2. 对社会责任的评价
本部分为工程项目建设中所对应阶段的社会责任情况调查
2. Evaluation of social responsibility
This part is divided into the corresponding stage of the project construction in order to investigate the performance of social responsibility in those stages.

2. 对社会责任的评价
本部分为工程项目建设中所对应阶段的社会责任情况调查
2. Evaluation of social responsibility
This part is divided into the corresponding stage of the project construction in order to investigate the performance of social responsibility in those stages.
[矩阵单选题] *

|  | 完全没有Don't have | 很少有Rare | 一般Normal | 偶尔有Often | 经常有Always |
| --- | --- | --- | --- | --- | --- |
| 项目立项阶段 Project initiating stage |  | | | | |
| 2.1 在项目立项阶段，政府提供了该项目给当地所带来的经济效益预测报告 During the project launch period, the government provided a forecast report on the economic benefits that the project would bring to the local area | ○ | ○ | ○ | ○ | ○ |
| 2.2 在项目立项阶段，政府考量了该项目对每个参与人员的经济方面影响 During the project initiation phase, The government considered the financial impact of the project on everyone involved | ○ | ○ | ○ | ○ | ○ |
| 2.3 在项目立项阶段，政府对项目的技术难度的可行性进行了分析 During the project launch period, the government analyzed the feasibility of the technical difficulties of the project | ○ | ○ | ○ | ○ | ○ |
| 2.4 在项目立项阶段，政府积极组织了公众参与，如公开听证会 During the project initiation phase, the government actively organized public participation, such as public hearings | ○ | ○ | ○ | ○ | ○ |
| 2.5 在项目立项阶段，政府考量了项目对环境、生态的影响，并且有环境评估报告（EIA） During the project initiation phase, The government considers the environmental and ecological impact of the project, and has the environmental assessment report (EIA) | ○ | ○ | ○ | ○ | ○ |
| 2.6 在项目立项阶段，政府考量了项目有尊重宗教、民族、文化的情况（如布施） In the project establishment stage, the government considers the situation that the project respects religion, nationality and culture (such as alms giving) | ○ | ○ | ○ | ○ | ○ |
| 2.7 在项目立项阶段，媒体公正的报道了与项目有关的宣传活动的合法性 During the project establishment phase, the media fairly reported the legitimacy of the publicity activities related to the project | ○ | ○ | ○ | ○ | ○ |
| 2.8 在项目立项阶段，媒体跟进了与项目有关的伦理道德及环境问题 During the project establishment stage, the media always paid attention to the ethical and environmental issues related to the project | ○ | ○ | ○ | ○ | ○ |
| 2.9 在项目立项阶段，媒体跟进了社区与公众的需求 During the project initiation stage, the media paid attention to the needs of the community and the public | ○ | ○ | ○ | ○ | ○ |
| 项目设计阶段 Project design stage |  | | | | |
| 2.10 在项目设计阶段，设计方在采用了在保证设计质量的情况下的经济最优的设计理念 In the design stage of the project, the designer adopts the economical optimal design concept under the condition of ensuring the design quality | ○ | ○ | ○ | ○ | ○ |
| 2.11 在项目设计阶段，设计方考量了项目在创新和科技进步上的情况 In the design stage of the project, the designer considers the project's innovation and technological progress | ○ | ○ | ○ | ○ | ○ |
| 2.12 在项目设计阶段，设计方积极使用了可持续设计及绿色设计理念 In the design stage of the project, the designer actively uses the concept of sustainable design and green design | ○ | ○ | ○ | ○ | ○ |
| 2.13 在项目设计阶段，设计方采用对以社区为中心的设计理念 In the design phase of the project, the designer adopts the concept of community-centered design | ○ | ○ | ○ | ○ | ○ |
| 2.14 在项目设计阶段，政府有公开项目的设计方案 During the project design stage, the government made public the project design scheme | ○ | ○ | ○ | ○ | ○ |
| 2.15 在项目设计阶段，政府有积极听取公众对设计方案提出的建议 During the design phase of the project, the Government actively listened to the public's suggestions on the design scheme | ○ | ○ | ○ | ○ | ○ |
| 2.16 在项目设计阶段，政府有对项目的设计成本进行监管 In the project design stage, the government supervises the design cost of the project | ○ | ○ | ○ | ○ | ○ |
| 项目建设阶段 Project construction stage |  | | | | |
| 2.17 在项目建设阶段，项目法人对工程质量和安全施工进行了监控 During the construction phase of the project, the project legal person shall monitor the project quality and safety construction | ○ | ○ | ○ | ○ | ○ |
| 2.18 在项目建设阶段，项目法人确保了资金安全及合理回报 During the construction phase of the project, the project legal person ensures the security of funds and reasonable returns | ○ | ○ | ○ | ○ | ○ |
| 2.19 在项目建设阶段，项目法人采纳了绿色建造理念 In the construction phase of the project, the project legal person adopts the concept of green construction | ○ | ○ | ○ | ○ | ○ |
| 2.20 在项目建设阶段，项目法人积极注重周边社区及公众的需求 2.20 During the construction phase of the project, the project legal person shall pay active attention to the needs of the surrounding communities and the public | ○ | ○ | ○ | ○ | ○ |
| 2.21 在项目建设阶段，承包商控制了工程的成本和工期 During the construction phase of the Project, the Contractor strictly controlled the cost and duration of the Project | ○ | ○ | ○ | ○ | ○ |
| 2.22 在项目建设阶段，承包商有对施工技术进行创新改进 During the construction phase of the project, the contractor innovates and improves the construction technology | ○ | ○ | ○ | ○ | ○ |
| 2.23 在项目建设阶段，承包商对施工阶段的资源进行了合理循环利用 During the construction phase of the project, the contractor has carried out reasonable recycling and utilization of resources in the construction phase | ○ | ○ | ○ | ○ | ○ |
| 2.24 在项目建设阶段，承包商遵守了法律法规及行业规范 During the construction phase of the Project, the Contractor complied with laws, regulations and industry norms | ○ | ○ | ○ | ○ | ○ |
| 2.25 在项目建设阶段，承包商确保了工程质量和施工安全 During the construction phase of the Project, the Contractor ensured the quality of the Works and construction safety | ○ | ○ | ○ | ○ | ○ |
| 2.26 在项目建设阶段，承包商对工地周边的社区、地区采取了生态环境保护措施 During the construction phase of the project, the contractor took measures to protect the ecological environment of the surrounding communities and areas | ○ | ○ | ○ | ○ | ○ |
| 2.27 在项目建设阶段，承包商有积极维护工地周边社区关系 During the construction phase of the project, the contractor actively maintains the community relations around the site | ○ | ○ | ○ | ○ | ○ |
| 2.28 在项目建设阶段，承包商有积极处理施工紧急公共事件 During the construction phase of the project, the contractor actively deals with emergency public events during construction | ○ | ○ | ○ | ○ | ○ |
| 2.29 在项目建设阶段，监理方有对工程质量和安全进行监督 During the construction phase of the project, the supervisor shall supervise the project quality and safety | ○ | ○ | ○ | ○ | ○ |
| 2.30 在项目建设阶段，监理方有对项目施工员工的权益监督 During the construction phase of the project, the supervisor shall supervise the rights and interests of the project construction staff | ○ | ○ | ○ | ○ | ○ |
| 2.31 在项目建设阶段，监理方有对项目的环境保护措施进行监督 During the construction phase of the project, the supervisor shall supervise the environmental protection measures of the project | ○ | ○ | ○ | ○ | ○ |
| 2.32 在项目建设阶段，供应商有保障施工材料的质量 In the construction phase of the project, the supplier shall guarantee the quality of construction materials | ○ | ○ | ○ | ○ | ○ |
| 2.33 在项目建设阶段，供应商有积极推广及使用绿色材料 In the construction phase of the project, the supplier actively promotes and uses green materials | ○ | ○ | ○ | ○ | ○ |
| 项目运营阶段 Project operating stage |  | | | | |
| 2.34 在项目运营阶段，运营商有对工程进行日常维护 During the operation phase of the project, The operator carries out routine maintenance on the project | ○ | ○ | ○ | ○ | ○ |
| 2.35 在项目运营阶段，运营商有控制工程的运营成本和质量 In the project operation stage, the operator shall control the operation cost and quality of the project | ○ | ○ | ○ | ○ | ○ |
| 2.36 在项目运营阶段，运营商有遵守法律、行业规范及合同约定 During the operation phase of the project, the operator shall comply with laws, industry norms and contract provisions | ○ | ○ | ○ | ○ | ○ |
| 2.37 在项目运营阶段，运营商有对资源进行合理利用 In the operation stage of the project, the operator makes reasonable use of resources | ○ | ○ | ○ | ○ | ○ |
| 2.38 在项目运营阶段，运营商有对社区、地区生态环境进行保护 In the project operation stage, the operator has to protect the community and regional ecological environment | ○ | ○ | ○ | ○ | ○ |
| 2.39 在项目运营阶段，运营商有积极维护与社区的关系 In the operation phase of the project, the operator actively maintains the relationship with the community | ○ | ○ | ○ | ○ | ○ |

3. 大众参与环境活动的程度
本部分考察的是您在项目的整个周期过程当中，做出的各种有利于环境保护的活动/行为的程度（不论活动大小），请根据您个人的感知以及实际情况来填写。

3. Stakeholder Environmental Activities Participation during the Project Life-cycle (SEAP-PL)
This section examines the various activities/behaviors that you have done in favor of environmental protection during the whole cycle of the project (regardless of activity size). Please fill in according to your personal perception.
[矩阵单选题] *

|  | 完全没有Don't have | 很少有Rare | 一般Normal | 偶尔有Often | 经常有Always |
| --- | --- | --- | --- | --- | --- |
| 3.1 有注意承包商/分包商是否使用了环保的建筑材料 There are concerns about the use of environmentally friendly building materials by contractors/subcontractors | ○ | ○ | ○ | ○ | ○ |
| 3.2 有注意承包商/分包商定是否期将垃圾堆放在指定位置 There is concern that the contractor/subcontractor will agree to place the garbage dump in the designated location | ○ | ○ | ○ | ○ | ○ |
| 3.3 有注意承包商/分包商是否将运输车辆重新干净后再上马路 There is concern about whether the contractor/subcontractor has cleaned the transport vehicles before driving on the road | ○ | ○ | ○ | ○ | ○ |
| 3.4 有注意项目相关人员是否定期参与环境保护教育 There are concerns about whether people involved in the project regularly participate in environmental protection education | ○ | ○ | ○ | ○ | ○ |
| 3.5 有注意政府是否在各个区域定点设定了垃圾桶 There are concerns about whether the government has targeted garbage bins in various areas | ○ | ○ | ○ | ○ | ○ |
| 3.6 有注意政府是否制定了保护环境政策及措施并实施，且在这个过程中我能学到很多环境保护知识 There are concerns about whether the government has formulated and implemented environmental protection policies and measures. In this process, I can learn a lot of environmental protection knowledge | ○ | ○ | ○ | ○ | ○ |
| 3.7 有注意政府、媒体等是否积极宣传环境保护的重要性，会给项目提出自己对环境保护的建议 There are concerns about whether the government media actively publicize the importance of environmental protection, and put forward my own suggestions on environmental protection to the project | ○ | ○ | ○ | ○ | ○ |
| 3.8 有注意媒体是否对环境保护措施做得好的企业或个人进行公开表彰 There are concerns about whether the media have publicly praised enterprises or individuals who have done well in environmental protection measures | ○ | ○ | ○ | ○ | ○ |
| 3.9 有注意项目法人、承包商等是否积极采用绿色设计，如使用太阳能、风能等 Pay attention to whether the project legal person and contractor actively adopt green design, such as solar energy and wind energy, etc | ○ | ○ | ○ | ○ | ○ |
| 3.10 给项目有关人员提出自己对他们所做的保护环境工作的看法 Give your opinion to the people involved in the project about what they are doing to protect the environment | ○ | ○ | ○ | ○ | ○ |
| 3.11 有注意群众自觉进行垃圾分类投放，不乱丢垃圾 There are concerns about people consciously classify garbage and do not litter | ○ | ○ | ○ | ○ | ○ |

4. 您对环境补偿措施的满意程度
本部分考察您们对环境补偿措施的满意程度，环境补偿措施指的是以保护生态环境、促进人与自然和谐为目的，调整生态环境保护和建设相关各方之间利益关系的一种制度安排，如：谁污染谁治理，谁破坏谁保护。请根据您的个人感知进行填写。

4. Satisfaction with the Environmental Compensation Mechanism (SECM)
This section examines the stakeholders' satisfaction with the environmental compensation mechanism. The environmental compensation measure refers to an institutional arrangement that adjusts the interest relations between the parties concerned in the ecological environmental protection and construction for the purpose of protecting the ecological environment and promoting the harmony between man and nature. For example, whoever pollutes will be responsible for the treatment, and whoever destroys will be responsible for the protection. Please fill in according to your personal perception.
[矩阵单选题] *

|  | 很不满意Unsatisfied at all | 不满意Unsatisfied | 一般Normal | 满意Satisfied | 很满意Very satisfied |
| --- | --- | --- | --- | --- | --- |
| 您对目前环境补偿措施的满意程度 Your satisfaction level of current environmental compensation measures |  | | | | |
| 4.1 公众自觉守护环境卫生 The public consciously protects environmental hygiene | ○ | ○ | ○ | ○ | ○ |
| 4.2 环保专家积极推动环保活动 Environmental experts actively promote environmental protection activities | ○ | ○ | ○ | ○ | ○ |
| 4.3 企业改进生产工艺、制造等技术减少对环境的污染 The enterprise shall improve the production process and manufacturing technology to reduce environmental pollution | ○ | ○ | ○ | ○ | ○ |
| 4.4 企业提升能源使用效率 Companies improve energy efficiency | ○ | ○ | ○ | ○ | ○ |
| 4.5 建立以政府投入为主、全社会支持生态环境建设的投资融资体制 An investment and financing system with government investment as the main component and the whole society supporting ecological environment construction shall be established | ○ | ○ | ○ | ○ | ○ |
| 4.6 政府支持替代能源的生产和使用 The government supports the production and use of alternative energy sources | ○ | ○ | ○ | ○ | ○ |
| 4.7 政府制定的环境保护税收政策 The government formulates tax policies for environmental protection | ○ | ○ | ○ | ○ | ○ |
| 4.8 研究者为完善环境补偿措施提供的科技和理论支撑 Scientific and theoretical support provided by researchers for improving environmental compensation measures | ○ | ○ | ○ | ○ | ○ |
